# On the assessment of the added value of new predictive biomarkers

**DOI:** 10.1186/1471-2288-13-98

**Published:** 2013-07-29

**Authors:** Weijie Chen, Frank W Samuelson, Brandon D Gallas, Le Kang, Berkman Sahiner, Nicholas Petrick

**Affiliations:** 1 Division of Imaging and Applied Mathematics, Office of Science and Engineering Laboratories, Center for Devices and Radiological Health, Food and Drug Administration, 10903 New Hampshire Avenue, Silver Spring, MD 20993, USA

**Keywords:** Biomarkers, Classification, Area under the ROC curve

## Abstract

**Background:**

The surge in biomarker development calls for research on statistical evaluation methodology to rigorously assess emerging biomarkers and classification models. Recently, several authors reported the puzzling observation that, in assessing the added value of new biomarkers to existing ones in a logistic regression model, statistical significance of new predictor variables does not necessarily translate into a statistically significant increase in the area under the ROC curve (AUC). Vickers et al. concluded that this inconsistency is because AUC “has vastly inferior statistical properties,” i.e., it is extremely conservative. This statement is based on simulations that misuse the DeLong et al. method. Our purpose is to provide a fair comparison of the likelihood ratio (LR) test and the Wald test versus diagnostic accuracy (AUC) tests.

**Discussion:**

We present a test to compare ideal AUCs of nested linear discriminant functions via an F test. We compare it with the LR test and the Wald test for the logistic regression model. The null hypotheses of these three tests are equivalent; however, the F test is an exact test whereas the LR test and the Wald test are asymptotic tests. Our simulation shows that the F test has the nominal type I error even with a small sample size. Our results also indicate that the LR test and the Wald test have inflated type I errors when the sample size is small, while the type I error converges to the nominal value asymptotically with increasing sample size as expected. We further show that the DeLong et al. method tests a different hypothesis and has the nominal type I error when it is used within its designed scope. Finally, we summarize the pros and cons of all four methods we consider in this paper.

**Summary:**

We show that there is nothing inherently less powerful or disagreeable about ROC analysis for showing the usefulness of new biomarkers or characterizing the performance of classification models. Each statistical method for assessing biomarkers and classification models has its own strengths and weaknesses. Investigators need to choose methods based on the assessment purpose, the biomarker development phase at which the assessment is being performed, the available patient data, and the validity of assumptions behind the methodologies.

## Background

Advances in genomics, proteomics, and high-throughput biotechnologies have generated many biomarkers with potential clinical value in diagnosis, assessment of prognosis, prediction of risk and therapy response, and many other applications. In a typical application, a classification model is used to combine multiple biomarkers (predictors) to predict a binary outcome such as diseased/nondiseased, responders/non-responders to therapy, etc. A particular problem of interest is to determine if a set of new biomarkers has added value. The added value is determined by building two nested classification models: 1) a partial model with the existing biomarkers, and 2) a full model with the new biomarkers combined with the existing biomarkers. One approach is to test the statistical significance of the new biomarkers with the binary outcome adjusted for the existing biomarkers in the full model, e.g., likelihood ratio (LR) test or Wald test in the logistic regression model. Another approach is to compare the diagnostic performance of the two nested models in terms of a diagnostic accuracy metric, for example, the widely used area under the receiver operating characteristic (ROC) curve or AUC.

Recently, several authors [1,2] reported the puzzling observation that the aforementioned two approaches are not always consistent with each other, namely, statistical significance of new predictor variables does not necessarily translate into a statistically significant increase in AUC. Vickers et al. [1] used simulation data to compare the LR test, the Wald test, and the DeLong et al. method for the comparison of AUC [3]. Their results showed that, under the null hypothesis (i.e., the new biomarkers are useless), the LR test and the Wald test both yield the nominal type I error rate whereas the DeLong test of AUC yields type I error that is far below the nominal level (i.e., it is extremely conservative). They also showed that the DeLong test of AUC is much less powerful than the other two tests under the alternative hypothesis. Demler et al. [2] reported similar simulation findings and the authors suggested that the DeLong approach should not be used for testing the null hypothesis but can be used for estimating the confidence interval of the difference of AUC values once the new biomarkers are shown to be significantly associated with the outcome.

We believe that the conclusions in Vickers et al. [1] may be misleading for practitioners. For example, they stated that “Although comparison of AUCs is a conceptually equivalent approach to the likelihood ratio and Wald test, it has vastly inferior statistical properties.” This conclusion statement is not substantiated because their simulation data are about a particular statistical method (namely the DeLong et al. method [3]) being used in a particular way (actually a misuse as we will show in this paper), but their conclusion is about the general AUC metric. Their conclusion inappropriately puts a negative aura on ROC analysis and paints AUC as ineffective. We will show in this paper that, given an appropriate statistic and distributional assumption, the hypothesis in the test of AUC can be equivalent to that in the LR or Wald tests and, for the particular statistic for the test of AUC we consider, the test of AUC actually can perform better than those statistical significance tests at small sample sizes.

In addition, in both the Vickers et al. paper [1] and the Demler et al. paper [2], the dataset that was used for training the model was also used as the test dataset, and the resulting resubstitution AUC values without and with the new biomarkers were compared using the DeLong et al. method. This is clearly a misuse of the DeLong method (as also pointed out in Demler et al. [2]) because the DeLong et al. method is designed for comparing two *fixed* models that are tested on a common dataset independent of the training set. Changing the model is not expected or accounted for in the DeLong et al. variance estimate. We will clarify the scope of the DeLong et al. method and show that the method has nominal error rates when used in its designed scope. We will also show that the hypothesis in the DeLong et al. method is different from those in the statistical significance tests of new predictor variables and therefore it cannot be compared with those tests.

Finally, we will summarize the pros and cons of the four methods we consider in this paper.

## Discussion

To facilitate our discussions, we begin with a brief review of the basic elements for the assessment of statistical learning models for pattern classification. We suppose that the model is trained with a finite training dataset *r* that has a size *N*≡*N*_0_+*N*_1_, where *N*_0_ and *N*_1_ are the number of observations sampled from the actually negative class and the actually positive class respectively. The diagnostic accuracy (e.g., AUC) estimated on an independent test set *t* by the nonparametric unbiased estimator is denoted as ${Â}_{\text{rt}}$. The AUC estimated by testing on the training set *r*, known as the resubstitution estimator, is denoted as ${Â}_{\text{rr}}$.

If we take the expectation of ${Â}_{\text{rt}}$ over the population of test sets conditional on a fixed and finite training set, denoted $A_{r}\equiv E_{t}\left[{Â}_{\text{rt}}|\text{training set}r\right]$, we have the performance of the fixed classification model. This conditional performance *A*_*r*_ is called the *true* performance by some authors (e.g., Efron [4]) in the sense that it is the performance that is truly expected for the population after the model is trained and released in the field.

When the training set is treated as random, *A*_*r*_ is a random variable and has uncertainty due to the randomness of the finite training set. The expectation of *A*_*r*_ over the population of training sets of the same size *N*≡*N*_0_+*N*_1_ is denoted as $Ā(N_{0},N_{1})=E_{r}\left[A_{r}\right]$. Note that Ā is a function of the training sample size and can be depicted in a “learning curve”. The ideal or theoretically optimal performance of the model is denoted as A≡Ā(∞,∞), i.e., the performance of the model when sizes of both the training set and the test set are infinity.

**Figure 1:**
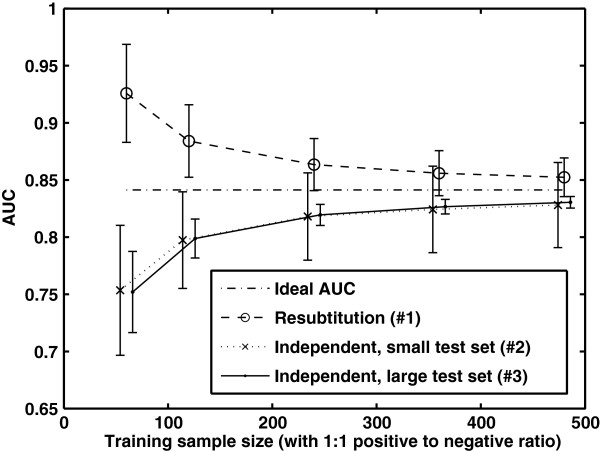
**“Antler” plot for the logistic regression model.** Fifteen simulated biomarkers are assumed to follow a pair of normal distributions for the two classes. At each training sample size, the AUC performance is estimated in one Monte Carlo (MC) trial with (#1) resubstitution, (#2) a small independent test set (60 observations per class), and (#3) a large independent test set (10,000 observations per class). The MC trial is repeated *independently* 1,000 times and the sample mean and the sample standard deviation (SD) of the estimated AUC values are calculated for each estimator. The figure plots the theoretically ideal AUC and the sample mean AUC (±1 SD) at training sample sizes 60, 120, 240, 360, and 480 (note that the plot is shifted a bit horizontally to avoid overlap between error bars).

Figure 1 shows a typical plot of the learning curves of a logistic regression model, which we call the “antler” plot for its typical shape. This plot was generated using simulated data. Fifteen simulated biomarkers are assumed to follow a pair of normal distributions for the two classes with user-designated parameters (the specific values are those of the first 15 variables in Table 1). At each training sample size, the AUC performance of the trained logistic regression model is estimated in one Monte Carlo (MC) trial with three estimators: (#1) resubstitution, (#2) a small independent test set (60 observations per class), and (#3) a large independent test set (10,000 observations per class). The MC trial is repeated *independently* 1,000 times retraining the models and testing them each time. The sample mean and the sample standard deviation (SD) of the estimated AUC values are computed for each of the three estimators. The figure plots the theoretically ideal AUC for the logistic regression model with the given distributions and parameters (horizontal line) and the sample mean AUC (±1 SD) of the three estimators as a function of the training sample size.

**Table 1:** User-selected mean and covariance matrix parameters for the normal distributions in simulating the joint distributions of 16 biomarkers

		**Null hypothesis**	**Alternative hypothesis**
Negative class	Mean	[ 0,…,0]_1×16_	[ 0,…,0]_1×16_
	Cov	Identity *I*_16×16_	Identity *I*_16×16_
Positive class	Mean	[ 0.7,0.6,0.6,0.5,0.5,0.3,0.3,0.2,…	[ 0.7,0.6,0.6,0.5,0.5,0.3,0.3,0.2,…
		0.2,0.1,0.1,0.1,0,0,0,0]	0.2,0.1,0.1,0.1,0,0,0,0.6]
	Cov	Identity *I*_16×16_	Identity *I*_16×16_
Ideal AUCs for LDF		0.8413 (15 biomarkers) vs.	0.8413 (15 biomarkers) vs.
		0.8413 (16 biomarkers)	0.8613 (16 biomarkers)

As illustrated in our simulation, with a finite training sample for every MC trial, the resubstitution estimator (#1) ${Â}_{\text{rr}}$ yields an optimistically biased estimate of the ideal performance of the model, and the independent validation estimators (#2, #3) ${Â}_{\text{rt}}$ yields a pessimistically biased estimate of the ideal performance *A*. In general, the variability of ${Â}_{\text{rt}}$ arises from both the finite training set and the finite test set (estimator (#2)), as shown in Figure 1. For estimator (#3), the test set is so large that the testing variability negligible. In this case, ${Â}_{\text{rt}}$ is *approximatelyA*_*r*_ and the error bar represents the variability *primarily* due to the randomness of the training set *r*, i.e., (#3) in Figure 1 is basically a plot of *A*_*r*_.

Bearing these basic characteristics in mind, we will be able to discuss different statistical tests and the corresponding target performance (*A* or *A*_*r*_).

### LR test, Wald test, and the F test of the ideal AUC for linear models

In this section, we consider how to decide whether a set of *q* new biomarkers have added value to *p* existing ones in two nested linear models. The ideal linear models (i.e., the theoretically true classification functions) can be written as: the partial model $h_{p}\;=\;\Sigma_{i=1}^{p}v_{i}x_{i}$ and the full model $h_{p+q}\;\;=\;\;\Sigma_{i=1}^{p}w_{i}x_{i}\;+\;\Sigma_{j=1}^{q}w_{p+j}x_{p+j}$. Statistical significance tests such as the LR test and the Wald test are readily available for the commonly used logistic regression model. In these tests, the null hypothesis is that the weight coefficients of the new biomarkers (*w*_*p*+1_,...,*w*_*p*+*q*_) are all zeros. The likelihood ratio expresses how many times more likely the data are under the full model than the partial model. Under the null hypothesis, the test statistic, which is twice the logarithm of the likelihood ratio, asymptotically follows a chi-square distribution with *q* degrees of freedom. The univariate (*q* = 1) Wald test statistic is the ratio of the maximum likelihood estimation of the weight coefficient ${ŵ}_{p+1}$ to an estimate of its standard error. The test statistic asymptotically follows a standard normal distribution under the hypothesis *w*_*p*+1_ = 0. More generally, the multivariate Wald test statistic can be defined analogously and it asymptotically follows a chi-square distribution with *q* degrees of freedom under the null hypothesis (for details, see Hosmer [5]).

Alternatively, one can test the null hypothesis that the ideal AUC for the partial model is equal to that for the full model, which we denote as $H_{0}\;:\;A(\;p)\;=\;A(\;p+q)$. Assuming that the two-class biomarker data follow a pair of multivariate normal distributions, the optimal linear model that maximizes the AUC is Fisher’s linear discriminant function (LDF) [6]. Then hypothesis $H_{0}$ is equivalent to $H_{0}^{\prime }:\;D(p)=D(p+q)$, where $D(n)=\sqrt{{\left(\mu_{1}-\mu_{2}\right)}^{T}V^{-1}(\mu_{1}-\mu_{2})}$ is the Mahalanobis distance between the normal distributions of the two classes for *n* biomarkers with mean vectors *μ*_1_, *μ*_2_ of length *n* respectively and a common *n*×*n* covariance matrix *V*. This equivalence is because of a monotonic one-to-one mapping between the ideal AUC for LDF and the Mahalanobis distance,

(1) $$A(n)=\Phi \left(\frac{D(n)}{\sqrt{2}}\right),$$

where *Φ* is the cumulative distribution function of the standard normal distribution.

Consequently the test of ideal AUC for two nested linear discriminant functions ($H_{0}$) can be achieved by the test of the Mahalanobis distance ($H_{0}^{\prime }$). Under the null hypothesis $H_{0}^{\prime }$, Rao [7] showed that the test statistic

(2) $$\begin{array}{ll} U\equiv & \frac{N_{0}+N_{1}-p-q-1}{q}\\ & \;\times \left(\;\frac{1+N_{0}N_{1}{\hat{D(\;p+q)}}^{2}\;/(N_{0}\;+\;N_{1})(N_{0}\;+\;N_{1}\;-\;2)}{1+N_{0}N_{1}{\hat{D(\;p)}}^{2}\;/(N_{0}\;+\;N_{1})(N_{0}\;+\;N_{1}-2)}-1\;\right) \end{array}$$

follows the F distribution with *q* and (*N*_0_+*N*_1_−*p*−*q*−1) degrees of freedom, where ${D\hat{\left(\;p+q\right)}}^{2}$ and ${\hat{D(\;p)}}^{2}$ are the sample Mahalanobis distances computed from a training sample consisting of *N*_0_ actually negative subjects and *N*_1_ actually positive subjects.

As shown by Delmer et al. [8], under the multivariate normality assumption, equality of two ideal AUCs for two nested linear discriminant functions is equivalent to discriminant coefficients equal to zero for variables not shared by the two models. This means that the LR test, the Wald test, and the F test actually test the same null hypothesis with different test statistics. However, the F test is an exact test, whereas the LR test and the Wald test are asymptotic tests. The main difference we expect is that the two types of methods may produce results of observable difference with finite samples. We provide a simulated example to compare the methods at various sample sizes.

In our simulation, we intend to assess if one new “biomarker” has added value to 15 existing ones. We do both a null-hypothesis experiment (i.e., the new biomarker has no added value) and an alternative-hypothesis experiment (i.e., the new biomarker has some added value). We assumed the simulated 16 biomarkers to follow a pair of multivariate normal distributions with parameters specified in Table 1 (the 16th is the new biomarker). These parameters were chosen by taking into account the following considerations (but otherwise arbitrary): (1) to simulate a set of biomarkers that have a variety of performance levels for a single biomarker and a medium performance level for the model; (2) to allow for useless existing biomarkers (false positives from previous studies); and (3) to be simple. The simulation may be unrealistic but only serves to demonstrate the properties of the significance testing methods.

Each experiment consisted of repeated independent MC trials. In each MC trial, we drew a training sample of a specified size with all the 16 biomarkers from the normal distributions with the designated parameters described above. We then performed the LR test and Wald test for the 16^*th*^ biomarker in the logistic regression model and also the F test of the ideal AUC. We called it a significant finding if the P value was less than 0.05. We repeated the trials 20,000 times and calculated the fraction of significant findings. The fraction is the observed type I error rate in the null-hypothesis experiment and the statistical power in the alternative-hypothesis experiment. We varied the training sample size to examine its effect on the performance of these tests.

Table 2 summarizes the simulation results. Since the multivariate normality assumption is satisfied, the F test of the ideal AUC can be regarded as the gold standard on theoretical grounds. The simulation results indeed confirmed that the method has the expected type I error rate of 0.05 at various sample sizes. The LR test and the Wald test were found to have inflated type I errors when the sample size was small, and the type I error converged to the expected nominal value asymptotically with the increasing sample size. The statistical power was found to be quite similar across all the three tests.

**Table 2:** Comparison of different statistical tests in assessing whether a new biomarker has added value

**Null hypothesis**	***N***_**0**_**=60**	***N***_**0**_**=120**	***N***_**0**_**=120**	***N***_**0**_**=240**	***N***_**0**_**=480**
**(Type I error)**	***N***_**1**_**=30**	***N***_**1**_**=60**	***N***_**1**_**=120**	***N***_**1**_**=120**	***N***_**1**_**=480**
LR test	0.1033	0.0668	0.0585	0.0600	0.0546
Wald Test	0.0781	0.0608	0.0546	0.0571	0.0538
F test of ideal AUC	0.0495	0.0482	0.0481	0.0522	0.0515
Alt. Hypothesis	*N*_0_=60	*N*_0_=120	*N*_0_=120	*N*_0_=240	*N*_0_=480
(Power)	*N*_1_=30	*N*_1_=60	*N*_1_=120	*N*_1_=120	*N*_1_=480
LR test	0.5956	0.8569	0.9539	0.9896	1.0000
Wald Test	0.5394	0.8453	0.9506	0.9889	1.0000
F test of ideal AUC	0.5196	0.8514	0.9538	0.9915	1.0000

Fraction of significant findings in 20,000 Monte Carlo trials at statistical significance cut-off of 0.05. On the top are the results for the null-hypothesis experiment where the fraction of significant findings is the observed type I error rate. At the bottom are the results for the alternative-hypothesis experiment where the fraction of significant findings is the statistical power.

The three statistical tests are comparable because the null hypotheses in these tests are equivalent. In addition, they all only involve the training data. The LR test and the Wald test involve the statistics in training the logistic regression model [5]. The F test of the ideal AUC involves using the training data to compute the sample Mahalanobis distances and it is equivalent to training and testing the linear discriminant function with the same dataset. However, as we will show in the next section, the DeLong et al. method [3] tests a different hypothesis involving only the test data in computation and hence should not be compared with the three statistical tests presented in this section.

### The DeLong et al. method for AUC comparison: its scope and appropriate use

The widely used DeLong et al. method [3] is designed to nonparametrically compare two correlated ROC curves. The decision scores underlying the two ROC curves, which the authors called “diagnostic tests”, can be two physical measurements or test scores of two classification models that are measured or *tested* on a common sample of patients. For assessing a model, the method assumes that the model is trained and *fixed*, and a nonparametric approach is used to estimate its diagnostic performance on the whole intended population (*A*_*r*_) using the *testing* scores of a sample of patients randomly and *independently* drawn from that population. It makes no assumption on the distributions of the biomarker data or how the model is trained whatsoever except that the training data is independent of the test data. The approach accounts for the variability of the performance that arises when a different random test sample is tested.

For comparing two models (including but not limited to two nested models), again the method assumes that the two models have been trained, fixed, and then tested on a common dataset that is randomly and independently drawn from the population. In estimating the variability of the AUC difference for hypothesis testing, the approach accounts for the variance of each AUC due to the finite test sample and the covariance due to the use of a common test set. In short, the DeLong method is designed to test the null hypothesis that the true performance of two fixed models are equal: $A_{r}^{\left(1\right)}=A_{r^{\prime }}^{\left(2\right)}$, where the superscripts 1, 2 denote two models under comparison, and the subscripts indicate that the training data sets may be different.

The test statistic in the DeLong et al. method is a *z* score defined as the difference of AUC divided by its standard error. Theoretically, the variance estimator that DeLong et al. used is not unbiased. We have investigated an unbiased version of the variance estimator based on U statistics [9] (see Appendix for a brief explanation). However, the difference between the DeLong et al. variance estimator and the unbiased one is so tiny that it is not of practical importance, as we will show below.

To simulate the null hypothesis, we modeled the test scores of two models as a bivariate binormal distribution with a common covariance matrix [ 1,*ρ*;*ρ*,1] and means of [ 0,0] and [ *μ*,*μ*] for the actually negative class and the actually positive class respectively. In each MC trial, we drew a sample of test scores from the designated distribution and applied the DeLong et al. method [3] and the U-statistics based method [9] to compare the AUC values. By only sampling test scores we are not retraining the model - it is fixed. We called it a significant finding if the P value was less than 0.05. We repeated the trials 20,000 times and calculated the fraction of significant findings, which is the observed type I error rate. The chosen *ρ* and *μ* parameters and the results are shown in Table 3. The results show that the type I error rate of the DeLong et al. method is close to the nominal value of 0.05. Although it is found to be slightly conservative compared to the U-statistics-based method, the difference is probably not of practical importance. We conclude from these simulations that, within its designed scope, the DeLong et al. method behaves nearly as expected in terms of the type I error.

**Table 3:** **Simulation results demonstrating the application of the DeLong et al. method [**[3]**] and the U-statistics based method [**[9]**] for comparing two fixed models under the null hypothesis**

		***N***_**0**_**=50*N***_**1**_**=50**	***N***_**0**_**=50*N***_**1**_**=100**	***N***_**0**_**=100*N***_**1**_**=200**
*ρ*=0	*μ*=0	0.0531/0.0547	0.0501/0.0507	0.0515/0.0521
	*μ*=1	0.0515/0.0527	0.0542/0.0558	0.0503/0.0511
	*μ*=1.5	0.0482/0.0498	0.0503/0.0512	0.0496/0.0500
*ρ*=0.6	*μ*=0	0.0507/0.0530	0.0488/0.0505	0.0515/0.0526
	*μ*=1	0.0457/0.0493	0.0501/0.0519	0.0485/0.0500
	*μ*=1.5	0.0453/0.0486	0.0444/0.0471	0.0501/0.0517

Fraction of significant findings in 20,000 Monte Carlo trials at statistical significance cut-off of 0.05 for the DeLong et al. method and the U-statistics based method respectively.

The DeLong et al. method was clearly used beyond its scope in Vickers et al. [1]. In their simulations, they drew a dataset to train the model in each MC repetition, which violated the fixed-model assumption. In addition, they tested the model with the training data, which violates the requirement in the DeLong et al. method that the test data is independent of the model. Moreover, the variance of the resubstitution AUC estimator is a totally different quantity from the variance estimated in the DeLong et al. method.

To appropriately use the DeLong et al. method with a single dataset, one needs to partition the dataset into two independent sets: a training set and a test set. After the models are trained with the training set and *fixed*, their performance can be compared by applying the DeLong et al. method to the test results. The method can be used to compare any two *fixed* models that are tested on a common independent test set.

It is important to note the difference between the DeLong et al. method and the significance testing methods investigated in the previous section for comparing nested models. The null hypothesis in the significance testing methods in the previous section is that the ideal AUC values (the AUC values of models with infinite training) are equal: A^**(1)**^**=A**^**(2)**^. However, the null hypothesis in the DeLong et al. method is that the AUC values of models *trained with a finite dataset* are equal: $A_{r}^{\left(1\right)}=A_{r^{\prime }}^{\left(2\right)}$. The two hypotheses are not equivalent (recall the learning curves and error bars in Figure 1).

### Pros and cons of different methodologies

We have considered four methods including two statistical association tests (the LR test and the Wald test) and two types of AUC tests (the F test of ideal AUC and the nonparametric test of the conditional AUC including the DeLong et al. method and the U-statistics based method). We categorize these tests into two paradigms. Paradigm 1 includes the LR test, the Wald test, and the F test of ideal AUC. They all target an unconditional population parameter (the theoretical weight coefficients or the ideal AUC). The null hypotheses of these tests are equivalent. Paradigm 2 includes the statistical test of the conditional AUC including the DeLong et al. method and the U-statistics based method. They target a conditional population parameter. The null hypothesis is *not* equivalent with those in Paradigm 1. We summarize the pros and cons of the different paradigms and statistical tests below. 

1. *The purpose of assessment*: Paradigm 1 makes a statistical judgment on whether the new biomarkers have added value, which is a binary decision and does not indicate how much the added value is. Paradigm 2 does provide estimate and confidence interval of the classification performance on the general population and the potential gain (effect size) of diagnostic accuracy by adding the new biomarkers, i.e., it provides information not only on whether the new biomarkers have added value but also how much the added value is.

2. *Required resources*: Paradigm 1 requires training data only. Paradigm 2 requires both the training and the independent test data.

3. *Assumptions on the multiple-biomarker measurement data*: The F test of ideal AUC has the strongest assumption that the biomarker data follow multivariate normal distributions. The assumption of the LR test and the Wald test is that of the logistic regression, i.e., the biomarker data follow the general exponential family of distributions [10], which is much weaker than the multivariate normality assumption. The DeLong et al. method and the U-statistics based method are nonparametric and make no distributional assumption on the biomarker data in computing the AUC and its variance. However, they do assume asymptotic normality of the non-parametric AUC estimator, which is true for a U-statistic estimator [11].

4. *Applicability*: The F test of ideal AUC is limited to the linear discriminant function applying to the multivariate normal data. The LR test and the Wald test are readily available for the logistic regression model but not necessarily for other classification models. The nonparametric AUC test can be used for any classification model on any data.

5. *Performance*: The F test of ideal AUC performs perfectly well when its assumption is satisfied. The LR test and the Wald test generally perform well but can have inflated type I error with limited sample size. The nonparametric AUC tests perform perfectly well or nearly so for comparing the true AUC values of two fixed models. However, to assess the usefulness of new biomarkers independent of the finite training dataset, one would typically need a large training set to obtain well-trained stable models for comparison.

The hypotheses in the two paradigms converge if there are *infinite* training data (or practically a sufficiently large training sample), which unfortunately is rarely the case in practical applications. The practical implication of Paradigm 2 is that one may not have a sufficiently large training sample or the intention to demonstrate that the ideal or theoretically optimal performance is realized. It is more common to show only that the conditional performance of a new model is acceptable or better than other existing solutions. When this is the case, the model can be shown to be useful (but not necessarily optimal) by evaluating on an independent test set even for a small training sample.

It should be emphasized again that the model in Paradigm 2 must be fixed and frozen after it is trained with a finite dataset. If the model is re-trained, it must be re-validated with a new test set. When a model is expected to undergo additional rounds of re-training and one wishes to fully quantify the variation of the performance then the variability must also be quantified with respect to the varying training samples [9]. In this scenario, the DeLong et al. test would not be appropriate.

## Summary

We have shown that there is nothing inherently less powerful or disagreeable about ROC analysis or the area under the ROC curve as a statistic for showing the usefulness of new biomarkers or classification models. The cause of the inconsistency puzzle between the statistical significance tests of nested models and AUC tests revealed in Vickers et al. [1] is that the DeLong et al. method was incorrectly applied to a set of data for which it was not intended.

In addition, it is not unexpected that estimating performance of a model using the same data that was used to train that model, i.e. resubstitution, can result in extremely biased results. Indeed Vickers et al. [1] are aware of this and point out “that the use of patient-specific predictors from the estimated model as data ensures that the estimated ROC curve is biased upwards,” and “that predictive models need to be validated in independent test sets, or minimally by using cross-validation techniques.” Yet the authors do neither of these things in their simulation, from which they still conclude that methods that utilize ROC/AUC have far less power than other methods and are not suitable for evaluating new biomarkers.

It is worth mentioning that several other authors have pointed out the inappropriateness of using the DeLong et al. method to compare the ideal AUC based on resubstitution AUC estimators, for example, Demler et al. [2] and Pepe et al. [12]. Pepe et al. [12] further investigated methods for variance estimation based on the bootstrap but found the performance unsatisfactory. The fundamental difficulty lies in the fact that the difference of resubstitution AUCs does *not* follow a normal distribution under the null hypothesis. This is evident from the empirical data shown in Demler et al. [2] and is confirmed by our own empirical data (not shown). A similar observation has been reported for the resubstitution estimator of other metrics, for instance, Kerr et al. [13] reported the nonnormality of the resubstitution estimator of the integrated discrimination improvement index. We have shown in this paper that statistical inference on the ideal AUC based on resubstitution AUC estimators can be performed via Rao’s F test of the Mahalanobis distance. However, this method requires the multivariate normality assumption. Further research is warranted for a more general approach to this problem.

Paradigm 2 is fundamentally different from Paradigm 1. First, it tests the hypothesis that the performance of the models conditional on a fixed and finite training set are the same ($A_{r}\equiv E_{t}\left[{Â}_{\text{rt}}|\text{training set}r\right]$). This conditional performance is important in the setting where we would like to know how a model is expected to perform when it is released into the field. Second, Paradigm 2 requires an independent test dataset. Given an independent test dataset, the U-statistic AUC is asymptotically normal [11] and the DeLong et al. method [3] is appropriate and effective. The DeLong et al. method uses the test dataset to estimate the AUC performance and its uncertainty due to the finite test set. This may appear to be less efficient than other methods that use all the available samples for training, as pointed out by Pepe et al. [12]. However, the statistical power is not comparable as these two paradigms test different hypotheses. Moreover, the independent training/testing approach provides more information than the all-for-training approach, i.e., it tells not only whether the new biomarkers have added value but also how much the added value is.

Nonetheless, we acknowledge that, for statistical inference on the AUC conditional on a fixed and finite training set, the split-sample strategy is not necessarily the best one in terms of the mean square error. We have previously shown using Monte Carlo simulations that resampling based strategies such as the bootstrap and cross-validation may outperform the split-sample approach [14,15]. However, the data-partition strategy is still a practical trade-off for many reasons (e.g., formal statistical inference methodologies for many resampling strategies are still not mature or widely available). This is a topic beyond the scope of this paper.

Vickers et al. [1] recommend only doing one statistical test instead of two in assessing the added value of new biomarkers. However, this recommendation is overly simplistic and our results and discussions indicate: 

1. Different methods have different assumptions and applicability as we have summarized above. It is therefore desirable to choose a statistical test by carefully considering the assumptions and applicability of all the available tests during the study design stage.

2. At the early phase of biomarker development, it might be desirable for investigators to perform two or more statistical tests because their agreement generally provides additional confidence in the conclusions. If two tests do not give similar results, then it provides an opportunity to investigate if the methods are applied correctly and/or if the assumptions are violated.

3. Biomarker development is a phased process [16-18]. At the early phase, the patient sample may be limited such that a simple linear model such as the logistic regression is most appropriate. The purpose at this phase is mainly to assess whether the new biomarkers are promising, but the limited data may not allow for reliable assessment of how much value they actually add. Potentially the statistical significance tests for new predictor variables could serve the purpose. The diagnostic accuracy can also be assessed typically in a cross-validation fashion. At the later phases of development and evaluation, however, a more complex classification model could be adopted and the primary purpose is to assess how much diagnostic accuracy the new biomarkers can add to existing ones. Then an independent training and testing of the model and a rigorous assessment of the added diagnostic accuracy (e.g., AUC) is necessary.

As we have summarized, each method has its own strengths and weaknesses, and one should choose methods to maximize the strengths and minimize the weaknesses. The one-statistical-test-fits-all concept is problematic since there is no perfect approach for every application. Investigators need to choose methods based on the assessment purpose, the biomarker development phase at which the assessment is being performed, the available patient data, and the validity of assumptions behind the methodologies, among other considerations.

## Appendix

Here we explain briefly, from the theoretical point of view, the difference between DeLong et al. [3] and Chen et al. [9] in estimating the variance of the nonparametric AUC estimate. The nonparametric estimate of AUC given the test scores of a test sample of *N*_0_ actually negative subjects (*X*_*i*_,*i*=1,...,*N*_0_) and *N*_1_ actually positive subjects (*Y*_*j*_,*j*=1,...,*N*_1_) is

$$Â=\frac{1}{N_{0}N_{1}}\Sigma_{\text{ij}}\psi (X_{i},Y_{j})$$

where

$$\psi (X_{i},Y_{j})=\left\{\begin{array}{ll} 1 & X_{i}<Y_{j}\\ 0.5 & X_{i}=Y_{j}\\ 0 & X_{i}>Y_{j} \end{array}\right).$$

Using the generalized U-statistics by Hoeffding [11], the variance of Â is

(3) $$\text{Var}(Â)=\frac{\left(N_{0}-1\right)}{N_{0}N_{1}}\xi_{01}+\frac{\left(N_{1}-1\right)}{N_{0}N_{1}}\xi_{10}+\frac{1}{N_{0}N_{1}}\xi_{11}$$

where

$$\begin{array}{lll} \xi_{01}=\; & E\left[\psi (X_{i},Y_{j})\psi (X_{i^{\prime }},Y_{j})\right]-A^{2},\;\;i\neq i^{\prime }\; & \;\\ \xi_{01}=\; & E\left[\psi (X_{i},Y_{j})\psi (X_{i},Y_{j^{\prime }})\right]-A^{2},\;\;j\neq j^{\prime }\; & \;\\ \xi_{11}=\; & E\left[\psi (X_{i},Y_{j})\psi (X_{i},Y_{j})\right]-A^{2}.\; & \; \end{array}$$

To obtain an estimator of the variance of Â, DeLong et al. [3] followed Sen’s structural components method [19] to estimate the terms *ξ*_01_ and *ξ*_10_ and ignored the second-order *ξ*_11_ term in Eq. 3. To obtain an unbiased estimator of Var(Â), Chen et al. [9] replaced *ξ*_01_, *ξ*_10_, and *ξ*_11_ with their unbiased estimators using U-statistics. Terms in the expression for the covariance of two AUC estimates are treated similarly.

## Abbreviations

ROC: Receiver operating characteristic; AUC: Area under the ROC curve; LR: likelihood ratio

## Competing interests

The authors declare that they have no competing interests.

## Authors’ contributions

The study was conceived and designed by WC, FWS, BDG, BS, and NP. The programming and simulations were carried out by WC, LK, and BS. WC drafted the manuscript and all co-authors contributed critical revisions. All authors read and approved the final manuscript.

## Pre-publication history

The pre-publication history for this paper can be accessed here:

http://www.biomedcentral.com/1471-2288/13/98/prepub
